# Enophthalmos Is Not Present in Horner Syndrome

**DOI:** 10.1371/journal.pmed.0020120

**Published:** 2005-04-26

**Authors:** Robert Daroff

**Affiliations:** **1**CASE School of MedicineCleveland, OhioUnited States of America

The case report by Nautiyal et al. [1] is an instructive reminder that the first episode of an acute painful Horner Syndrome should prompt imaging of the ipsilateral internal carotid artery, since carotid dissection (as well as other conditions, such as high-grade stenosis) needs to be ruled out. Unfortunately, the authors perpetuate the extremely common misconception that enophthalmos accompanies ptosis and miosis in human Horner Syndrome. It is only an illusion of enophthalmos caused by the ptosis. This is evident in the left eye of their patient in Figure 1 of the case report.

Actual measurement with exophthalmometry clearly demonstrates the lack of enophthalmos. As stated by Loewenfeld ([2], p. 1139), “Animals such as cats, rats, or dogs have enophthalmos on the side of the sympathetic lesion. But in man, the enophthalmos is only apparent. The small palpebral fissure makes the eye look sunken in on the affected side, but the position of the globe in the orbit remains virtually unchanged. This has been found by all workers who have measured the supposed enophthalmos objectively.” Loewenfeld cites four supportive references.

Thompson and Miller ([3], p. 964) provide four additional references that the enophthalmos “is apparent rather than real.”
